# Impact of heparin-to-bivalirudin bridging time on hospitalization and safety outcomes in PCI for acute coronary syndrome

**DOI:** 10.3389/fcvm.2026.1752565

**Published:** 2026-04-13

**Authors:** Guiping Wang, Bolu Yang, Ruolin Zhang, Xiaokun Liu

**Affiliations:** 1 Tangshan Gongren Hospital, Tangshan, Hebei, China; 2 Global Health Research Center, Duke Kunshan University, Kunshan, Jiangsu, China

**Keywords:** acute coronary syndrome, anticoagulation therapy, bivalirudin, heparin, percutaneous coronary intervention

## Abstract

**Background:**

Bivalirudin is increasingly used during percutaneous coronary intervention (PCI) for acute coronary syndrome (ACS), but the optimal interval between discontinuing unfractionated heparin (UFH) and initiating bivalirudin remains unclear. This study examined the association between heparin-to-bivalirudin bridging time and clinical outcomes.

**Methods:**

This retrospective cohort included 197 ACS patients undergoing PCI. Bridging intervals were evaluated using two predefined thresholds: Scenario A (≤30 vs. >30 min) and Scenario B (≤20 vs. >20 min). Clinical, laboratory, and procedural data were collected. Outcomes included bleeding events, cardiovascular events, rehospitalization, and composite adverse outcomes. Multivariable logistic regression and binomial regression with generalized estimating equations were performed, and restricted cubic spline analyses assessed non-linear associations.

**Results:**

In Scenario A, hospitalization occurred significantly less frequently in the ≤30-min group compared with the >30-min group (8.9% vs. 21.6%). After adjustment, longer intervals were associated with higher hospitalization risk (OR = 3.20, 95% CI: 1.20–8.54; RR = 2.55, 95% CI: 1.15–5.66). No differences were observed for bleeding, cardiovascular events, or composite outcomes. In Scenario B, no significant associations were identified for any outcomes. Spline analyses revealed no dose-response relationship between bridging time and clinical outcomes.

**Conclusions:**

A heparin-to-bivalirudin bridging interval of ≤30 min may reduce hospitalization without increasing bleeding or cardiovascular events, whereas a 20-min threshold offers no clinical advantage. Bridging time may represent a modifiable procedural parameter that warrants further evaluation in prospective randomized trials.

**Clinical Trial Registration:**

ChiCTR2400089671.

## Introduction

Acute coronary syndrome (ACS) remains a leading cause of cardiovascular morbidity and mortality worldwide, with major contributing factors including hypertension, dyslipidemia, diabetes, and other cardiometabolic risk factors (1–4). Percutaneous coronary intervention (PCI) has become the cornerstone of ACS management, effectively restoring coronary blood flow and improving clinical outcomes (5). However, the mechanical disruption of atherosclerotic plaques during PCI triggers platelet activation and thrombin generation, potentially precipitating acute thrombotic complications (6, 7). Consequently, optimal periprocedural anticoagulation is fundamental to successful PCI outcomes. Unfractionated heparin (UFH) has historically been the anticoagulant of choice, offering well-established efficacy and favorable cost-effectiveness (8). Nevertheless, UFH has notable limitations, including unpredictable pharmacokinetics, the requirement for frequent monitoring, and risks of heparin-induced thrombocytopenia and heparin resistance (9, 10).

Bivalirudin, a synthetic direct thrombin inhibitor, has emerged as a compelling alternative, offering more predictable anticoagulant effects, rapid onset and offset of action, and reduced risk of thrombocytopenia compared to UFH (11–14). Clinical trials have established bivalirudin as an effective anticoagulant for patients with ACS undergoing PCI, with evidence suggesting comparable or superior efficacy in reducing ischemic events and substantially lower bleeding rates compared with heparin-based regimens (15, 16). However, the substantially higher acquisition cost of bivalirudin compared to UFH presents a significant economic barrier to its widespread adoption (17). A bridging strategy using initial UFH during diagnostic angiography, followed by bivalirudin only when PCI is necessary, may offer substantial economic advantages while maintaining clinical efficacy (18). Recent evidence has demonstrated the safety and efficacy of heparin-to-bivalirudin bridging in elective PCI settings (19), yet the optimal bridging interval between heparin discontinuation and bivalirudin initiation remains undefined.

Understanding the relationship between bridging duration and clinical outcomes is essential to establish evidence-based protocols that optimize both safety and cost-effectiveness. An excessively short bridging time may result in overlapping anticoagulant effects, potentially increasing bleeding risk, whereas a prolonged interval could lead to inadequate anticoagulation and thrombotic complications. Therefore, this retrospective cohort study aimed to investigate the association between heparin-to-bivalirudin bridging time and clinical outcomes, including bleeding events, cardiovascular events, and hospitalization rates, in patients with ACS undergoing PCI, with the goal of identifying an optimal bridging interval that balances therapeutic efficacy with economic considerations.

## Methods

### Study population

This study included patients diagnosed with ACS who underwent PCI at Tangshan Workers' Hospital between January 1, 2019, and December 31, 2021. Eligible patients were aged 18–85 years, with no gender restrictions. All participants or their legally authorized representatives provided written informed consent after being informed of the treatment plan.

Exclusion criteria comprised severe dysfunction of vital organs (liver or kidneys), coagulopathy or hematological disorders, known contraindications to study medications, and refusal to participate or provide informed consent. Additionally, patients with incomplete data on post-discharge activated partial thromboplastin time (APTT), post-discharge platelet counts, or cardiovascular and rehospitalization outcomes were excluded from the final analysis. The study protocol was approved by the Ethics Committee of Tangshan Workers' Hospital (Ethical Application Ref: GRYY-LL-KJ2021-K67).

### Grouping strategy and definitions

Participants were stratified based on the bridging interval between sodium heparin and bivalirudin discontinuation. Two threshold scenarios were predefined for analysis. Scenario A (30-min threshold): Group 1 consisted of patients with bridging intervals ≤30 min, while Group 2 included those with bridging intervals >30 min. Scenario B (20-min threshold): Group 1 comprised patients with bridging intervals ≤20 min, and Group 2 included those with bridging intervals >20 min.

The bridging time was defined as the interval between the last administration of sodium heparin and the initiation of bivalirudin during PCI.

### Anticoagulation protocol

All patients received standardized anticoagulation therapy. Before coronary angiography, patients received intravenous sodium heparin at 70–100 U/kg. Bivalirudin was administered with a loading dose of 0.75 mg/kg as an intravenous bolus, followed by continuous infusion at 1.75 mg/kg/h during the procedure and continued for up to 4 h post-procedure. For patients receiving glycoprotein IIb/IIIa inhibitors (GPI), heparin dosing was appropriately reduced according to clinical guidelines.

### Data collection

Comprehensive demographic and clinical data were collected at admission, including age, gender, education level, occupation, income, and anthropometric measurements (height, weight). Lifestyle factors, including, smoking history, and alcohol consumption, were documented. Medical history encompassing comorbidities (hypertension, diabetes mellitus), medication use (antidiabetic, lipid-lowering, aspirin, and antihypertensive medications), and prior cardiovascular events were recorded.

Laboratory tests were performed at admission and post-discharge, including complete blood count with platelet count, coagulation profile (APTT, and activated clotting time [ACT]), comprehensive metabolic panel (liver and renal function), lipid profile (triglycerides, total cholesterol), creatinine clearance rate, N-terminal pro-B-type natriuretic peptide (NT-proBNP), and cardiac biomarkers. GRACE and CRUSADE risk scores were calculated for all patients.

### Procedural data

Details of the PCI procedure were meticulously recorded, including surgical approach (radial or femoral), location and number of vascular lesions, number of stents implanted, contrast agent volume, TIMI blood flow classification before and after intervention, and precise timing of anticoagulant medication administration. ACT was measured immediately post-PCI and at 30 min, 1 h, and 2 h after discontinuing bivalirudin in both groups.

Post-discharge platelet counts and APTT were measured and recorded. Patients were followed for adverse outcomes, including bleeding events, major adverse cardiovascular events (MACE), all-cause mortality, nonfatal acute myocardial infarction, hospitalization events (defined as any re-hospitalization occurring within six months or one-year post-discharge), severe heart failure, malignant arrhythmias, acute target vessel revascularization, thrombocytopenia, and acute stent thrombosis. Adverse events were defined as a composite of cardiovascular events, death, and hospitalization events. Cardiovascular events encompassed acute myocardial infarction, severe heart failure, malignant arrhythmias, and acute target vessel revascularization.

### Statistical analysis

Continuous variables were assessed for normality using the Shapiro–Wilk test and visual inspection of histograms. Normally distributed continuous variables were presented as mean ± standard deviation (SD) and compared between groups using Student's *t*-test or analysis of variance. Categorical variables were presented as frequencies and percentages and compared using the chi-square test.

Preoperative and postoperative platelet counts and APTT values were compared between groups using Student's *t*-test. The mean differences in platelet counts and APTT from preoperative to postoperative periods were calculated for each group and compared using Student's *t*-test. Mean differences with 95% confidence intervals (CIs) were reported.

To examine potential non-linear associations between bridging time (as a continuous variable) and clinical outcomes, restricted cubic spline regression with four knots placed at the 5th, 50th, and 95th percentiles was performed. Models were adjusted for gender, age, aspirin use, preoperative platelet count, and number of stents implanted. Overall *p*-values for the association and *p*-values for non-linearity were calculated using likelihood ratio tests.

Clinical outcomes were analyzed using two complementary regression approaches. Model A (Logistic Regression): Binary outcomes were analyzed using multivariable logistic regression to estimate odds ratios (ORs) and 95% CIs for the association between bridging time groups and adverse outcomes. Model B (Binomial Regression with generalized estimating equations [GEE]): To account for potential within-patient correlation and provide risk estimates on the relative scale, GEE with a binomial distribution and log link function were employed to estimate rate ratios (RRs) and 95% CIs. This method supplements logistic regression by providing effect estimates at different scales and is robust to incorrect specification of the correlation structure. Both models were applied to analyze adverse events (composite outcome), cardiovascular events, death, hospitalization events, and bleeding events separately in Scenarios A and B. In addition, Model 1 were adjusted for age and gender. Model 2 was further adjusted for aspirin use, preoperative platelet count, and the number of stents implanted based on the model 1.

All statistical tests were two-sided, with *p*-values <0.05 considered statistically significant. Statistical analyses were performed using Stata version 17.0 (StataCorp, College Station, TX, USA) and R version 4.3.2.

## Results

### Study population and baseline characteristics

**Figure 1:**
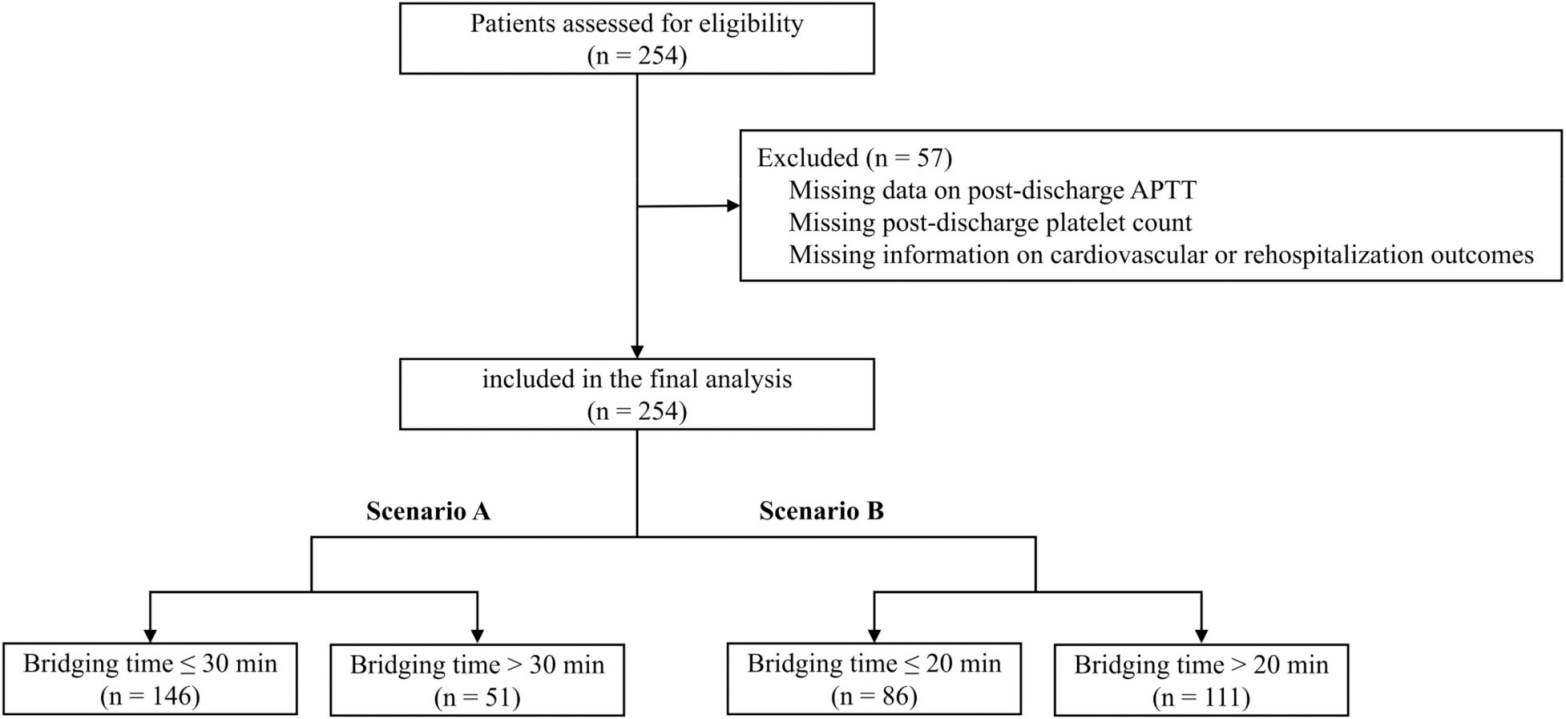
Flow diagram of participant selection and grouping.

Of the 254 patients initially assessed for eligibility, 57 were excluded due to missing data on post-discharge APTT, post-discharge platelet counts, or cardiovascular and rehospitalization outcomes. This resulted in a final analytical cohort of 197 patients (Figure 1). Patients were stratified into two groups based on bridging time thresholds in two predefined scenarios.

In Scenario A (30-min threshold), 146 patients had bridging times ≤30 min (Group 1) and 51 patients had bridging times >30 min (Group 2). The overall cohort had a mean age of 65.15 ± 9.47 years, with males comprising the majority. Baseline clinical characteristics, including triglyceride levels, total cholesterol, creatinine clearance rates, and comorbidities, were comparable between groups, with no statistically significant differences observed (all *p* > 0.05).

In Scenario B (20-min threshold), 86 patients were assigned to Group 1 (bridging time ≤20 min) and 111 to Group 2 (bridging time >20 min). The distribution of baseline demographic and clinical characteristics remained balanced between groups in this scenario as well (Table 1).

**Table 1:** Baseline characteristics of patients by bridging time across scenarios A and B.

Characteristics	Scenario A			Scenario B		
	Group 1	Group 2	*P*	Group 1	Group 2	*P*
*n*	146	51	—	86	111	—
Age (mean ± SD, years)	64.7 (9.8)	66.5 (8.5)	0.249	63.9 (10.2)	66.2 (8.8)	0.092
Male	110 (75.3)	32 (62.7)	0.084	64 (74.4)	78 (70.3)	0.520
BMI (mean ± SD, kg/m^2^)	25.2 (3.1)	25.1 (3.7)	0.837	25.1 (3.1)	25.20 (3.3)	0.853
Triglycerides (mean ± SD, mmol/L)	2.0 (2.5)	1.5 (0.6)	0.186	2.0 (2.8)	1.7 (1.6)	0.400
Total cholesterol (mean ± SD, mmol/L)	9.1 (56.0)	4.1 (1.0)	0.526	4.6 (1.2)	10.3 (64.2)	0.412
Creatinine clearance rate (mean ± SD, mL/min)	89.2 (29.0)	84.1 (270)	0.266	87.3 (31.5)	88.3 (26.2)	0.801
Antidiabetic medication	27 (18.5)	8 (15.7)	0.652	14 (16.3)	21 (18.9)	0.631
Lipid-lowering medication	61 (41.8)	17 (33.3)	0.288	34 (39.5)	44 (39.6)	0.988
Aspirin medication	66 (45.2)	21 (41.2)	0.618	36 (41.9)	51 (45.9)	0.567
Antihypertension medication	59 (40.4)	21 (41.2)	0.924	34 (39.5)	46 (41.4)	0.787
Alcohol consumption	34 (23.3)	9 (17.6)	0.401	19 (22.1)	24 (21.6)	0.937
Smoking	47 (32.2)	12 (23.5)	0.245	27 (31.4)	32 (28.8)	0.696
Hypertension	81 (55.5)	29 (56.9)	0.864	48 (55.8)	62 (55.9)	0.995
Diabetes	29 (19.9)	10 (19.6)	0.969	15 (17.4)	24 (21.6)	0.465
Number of stents implanted in the surgery (>2)	49 (33.6)	20 (39.2)	0.466	27 (31.4)	42 (37.8)	0.347

Unless otherwise indicated, data are presented as *n* (%). Scenario A and Scenario B were defined based on bridging times of 30 min and 20 min, respectively.

### Platelet counts and APTT

In Scenario A, Group 1 demonstrated significantly higher preoperative platelet counts compared to Group 2 (215.6 ± 61.6 vs. 196.9 ± 61.6 mmol/L; mean difference 18.69 mmol/L, 95% CI: 0.13–37.25; *p* = 0.049). This difference persisted postoperatively, with Group 1 maintaining higher platelet counts than Group 2 (204.7 ± 61.3 vs. 185.6 ± 38.4 mmol/L; mean difference 19.14 mmol/L, 95% CI: 1.08–37.20; *p* = 0.038). The mean change in platelet count from preoperative to postoperative values did not differ significantly between groups (*p* = 0.943).

Preoperative APTT was significantly higher in Group 1 than in Group 2 (45.0 ± 48.5 vs. 28.7 ± 43.5 s; mean difference 16.32 s, 95% CI: 1.15–31.48; *p* = 0.035), and this difference was amplified postoperatively (70.4 ± 68.8 vs. 41.5 ± 56.0 s; mean difference 28.97 s, 95% CI: 7.89–50.06; *p* = 0.007). However, the mean change in APTT from pre- to postoperative periods was not significantly different between groups (*p* = 0.284).

In Scenario B, similar patterns emerged. Group 1 exhibited significantly higher preoperative platelet counts (222.2 ± 69.5 vs. 202.0 ± 46.3 mmol/L; mean difference 20.21 mmol/L, 95% CI: 3.89–36.52; *p* = 0.016) and postoperative platelet counts (213.3 ± 70.1 vs. 189.3 ± 41.2 mmol/L; mean difference 24.06 mmol/L, 95% CI: 8.30–39.83; *p* = 0.003) compared to Group 2. Preoperative APTT was also significantly higher in Group 1 (50.6 ± 52.2 vs. 33.0 ± 42.5 s; mean difference 17.83 s, 95% CI: 4.52–31.13; *p* = 0.009), although this difference was not sustained postoperatively (69.3 ± 67.5 vs. 58.0 ± 66.1 s; *p* = 0.240). The mean change in APTT values remained comparable between groups (*p* = 0.532) (Table 2).

**Table 2:** Comparison of preoperative and postoperative platelet counts and APTT between groups in scenarios A and B.

Variables (mmol/L)	Scenario A				Scenario A			
	Group 1	Group 2	Diff (95% CI)	*P*	Group 1	Group 2	Diff (95% CI)	*P*
Preoperative Platelet Count	215.6 ± 61.6	196.9 ± 61.6	18.69 (0.13, 37.25)	0.049	222.2 ± 69.5	202.0 ± 46.3	20.21 (3.89, 36.52)	0.016
Postoperative Platelet Count	204.7 ± 61.3	185.6 ± 38.4	19.14 (1.08, 37.20)	0.038	213.3 ± 70.1	189.3 ± 41.2	24.06 (8.30, 39.83)	0.003
Mean Difference of Platelet Count*	10.9 ± 39.9	11.4 ± 37.0	−0.45 (−13.02, 12.11)	0.943	8.9 ± 43.4	12.7 ± 35.5	−3.85 (−14.94, 7.23)	0.493
Preoperative APTT	45.0 ± 48.5	28.7 ± 43.5	16.32 (1.15, 31.48)	0.035	50.6 ± 52.2	33.0 ± 42.5	17.83 (4.52, 31.13)	0.009
Postoperative APTT	70.4 ± 68.8	41.5 ± 56.0	28.97 (7.89, 50.06)	0.007	69.3 ± 67.5	58.0 ± 66.1	11.29 (−7.61, 30.20)	0.240
Mean Difference of APTT*	−25.4 ± 77.4	−12.8 ± 55.9	12.66 (−35.91, 10.59)	0.284	−18.4 ± 79.1	−25.0 ± 67.2	6.53 (−14.04, 27.11)	0.532

Scenario A and Scenario B were defined based on bridging times of 30 min and 20 min, respectively.

* Mean difference indicates the variation of platelet count and APTT before and after the surgery.

### Association between bridging time and clinical outcomes

**Figure 2:**
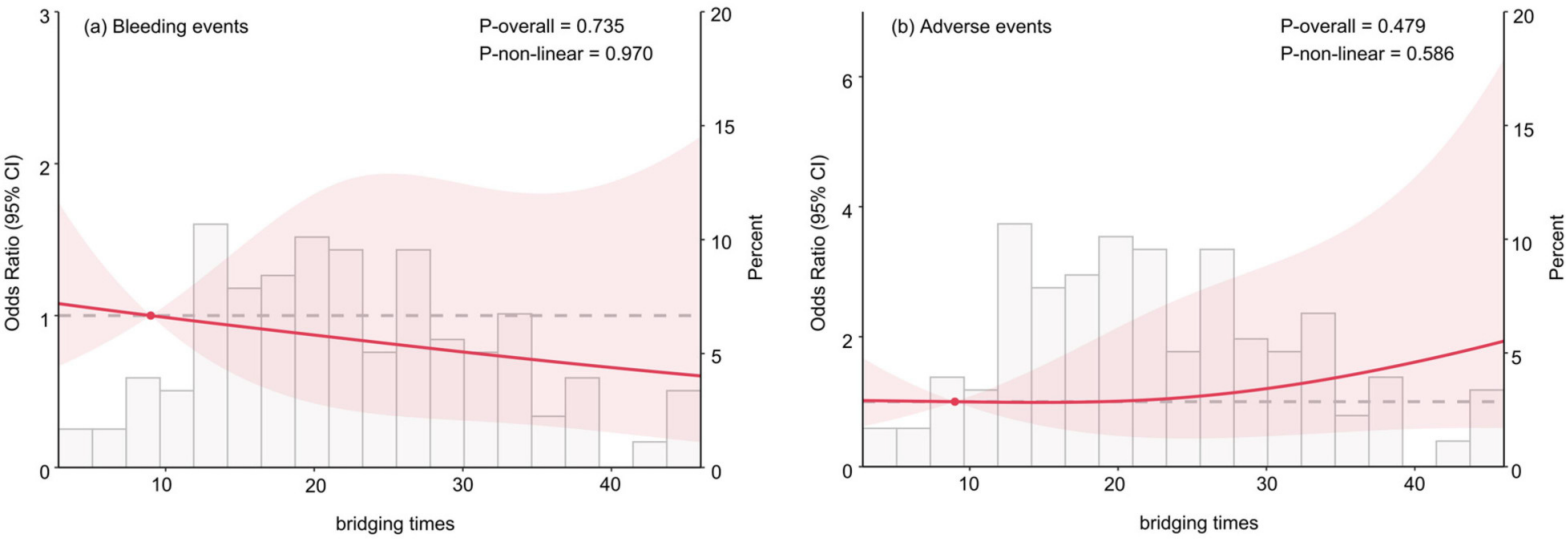
Association of the bridging times and the risk of bleeding and adverse events. **(a)** Bleeding events; **(b)** adverse events. The model was adjusted for gender, age, aspirin use, Preoperative Platelet Count, and the number of stents implanted.

Restricted cubic spline analysis, adjusted for gender, age, aspirin use, preoperative platelet count, and number of stents implanted, revealed no significant non-linear or linear association between bridging time (as a continuous variable) and the risk of bleeding events (overall *p* = 0.735, non-linear *p* = 0.970) or adverse events (overall *p* = 0.479, non-linear *p* = 0.586) (Figure 2).

### Clinical outcomes

In Scenario A, hospitalization events occurred less frequently in Group 1 compared to Group 2 (8.9% vs. 21.6%). After adjusting for gender, age, aspirin use, preoperative platelet count, and number of stents implanted (Model 2), logistic regression analysis (Model A) revealed a significant association, with Group 2 having 3.20 times higher odds of hospitalization compared to Group 1 (OR: 3.20, 95% CI: 1.20–8.54; *p* = 0.020). This association remained significant when analyzed using binomial regression with generalized estimating equations (Model B), which demonstrated a 2.55-fold increased rate of hospitalization in Group 2 (RR: 2.55, 95% CI: 1.15–5.66; *p* = 0.021).

Other adverse outcomes, including overall adverse events (16.4% vs. 21.6%), did not differ significantly between groups in Model 2 (Model A: OR: 1.75, 95% CI: 0.72–4.28, *p* = 0.217; Model B: RR: 1.36, 95% CI: 0.68–2.71, *p* = 0.379). Similarly, bleeding events (18.5% vs. 11.8%) showed no significant differences in the adjusted analyses (Model A: OR: 0.53, 95% CI: 0.19–1.45, *p* = 0.214; Model B: RR: 0.59, 95% CI: 0.27–1.31, *p* = 0.194). Cardiovascular events occurred in 7.5% of Group 1% and 0% of Group 2; statistical modeling was not feasible due to zero events in Group 2. One death (0.8%) occurred in Group 1, with no deaths recorded in Group 2; formal comparison was precluded by the sparse event data.

In Scenario B, no significant differences were observed between groups for any clinical outcomes after adjusting for the all covariates. Adverse events occurred in 17.4% of Group 1 and 18.0% of Group 2, with neither logistic regression (Model A: OR: 1.17, 95% CI: 0.52–2.64, *p* = 0.697) nor binomial regression (Model B: RR: 1.15, 95% CI: 0.63–2.13, *p* = 0.648) demonstrating significant associations. Hospitalization events (9.3% vs. 14.4%) showed no significant differences in either Model A (OR: 1.86, 95% CI: 0.71–4.91, *p* = 0.207) or Model B (RR: 1.68, 95% CI: 0.77–3.65, *p* = 0.189). Similarly, bleeding events (18.6% vs. 15.3%) and cardiovascular events (8.1% vs. 3.6%) did not differ significantly between groups in either analytical approach (Table 3).

**Table 3:** Comparison of Major events between groups in scenarios A and B.

			Model A*		Model B*	
Outcomes	Group 1	Group 2	Odds ratio (95% CI), *P* value		Rate ratio (95% CI), *P* value	
			Model 1	Model 2	Model 1	Model 2
Scenario A						
Adverse events^#^	24 (16.4%)	11 (21.6%)	1.46 (0.65, 3.27), 0.362	1.75 (0.72, 4.28), 0.217	1.37 (0.71, 2.63), 0.349	1.36 (0.68, 2.71), 0.379
Cardiovascular events	11 (7.5%)	0 (0%)	NA	NA	NA	NA
Death	1 (0.8%)	0 (0%)	NA	NA	NA	NA
Hospitalization events^¶^	13 (8.9%)	11 (21.6%)	2.94 (1.20, 7.18), 0.018	3.20 (1.20, 8.54), 0.020	2.46 (1.14, 5.29), 0.021	2.55 (1.15, 5.66), 0.021
Bleeding events	27 (18.5%)	6 (11.8%)	0.50 (0.19, 1.32), 0.160	0.53 (0.19, 1.45), 0.214	0.56 (0.25, 1.27), 0.165	0.59 (0.27, 1.31), 0.194
Scenario B						
Adverse events^#^	15 (17.4%)	20 (18.0%)	1.04 (0.50, 2.20), 0.909	1.17 (0.52, 2.64), 0.697	1.04 (0.57, 1.92), 0.893	1.15 (0.63, 2.13), 0.648
Cardiovascular events	7 (8.1%)	4 (3.6%)	0.39 (0.11, 1.41), 0.150	0.42 (0.10, 1.83), 0.249	0.42 (0.13, 1.35), 0.145	0.45 (0.10, 1.91), 0.276
Death	1 (1.2%)	0 (0%)	NA	NA	NA	NA
Hospitalization events^¶^	8 (9.3%)	16 (14.4%)	1.71 (0.69, 4.25), 0.249	1.86 (0.71, 4.91), 0.207	1.59 (0.70, 3.61), 0.266	1.68 (0.77, 3.65), 0.189
Bleeding events	16 (18.6%)	17 (15.3%)	0.71 (0.33, 1.52), 0.373	0.61 (0.27, 1.39), 0.240	0.76 (0.42, 1.38), 0.364	0.66 (0.35, 1.27), 0.213

Model 1 were adjusted for age and gender. Model 2 was further adjusted for aspirin use, preoperative platelet count, and the number of stents implanted based on the model 1.

Scenario A and Scenario B were defined based on bridging times of 30 min and 20 min, respectively.

* Model A utilized logistic regression, while Model B employed generalized estimating equations (GEE).

^#^ Adverse events include cardiovascular events, death, and hospitalization.

^¶^ Hospitalization refers to any re-hospitalization occurring within six months or one year.

## Discussion

This retrospective observational study investigated the association between heparin-to-bivalirudin bridging time and clinical outcomes in patients with ACS undergoing PCI. Our principal finding was that patients with bridging intervals ≤30 min (Scenario A) were associated with a lower risk of hospitalization compared to those with longer intervals, with no apparent increase in bleeding or other adverse events. However, this association was not observed when the bridging threshold was reduced to 20 min (Scenario B), and restricted cubic spline analysis revealed no dose-response relationship between bridging time as a continuous variable and clinical outcomes.

Our findings align with and extend previous research on anticoagulation strategies in PCI. Large-scale trials such as ACUITY (20) and HORIZONS-AMI (21) established that bivalirudin provides comparable antithrombotic efficacy to heparin while significantly reducing bleeding complications in patients with ACS undergoing PCI. However, these landmark studies primarily compared bivalirudin monotherapy with heparin-based regimens and did not examine periprocedural bridging strategies or the timing of anticoagulant transitions. Similarly, Li et al. (22) demonstrated the safety and effectiveness of bivalirudin vs. heparin in elective PCI during long-term follow-up, but their analysis also did not address differences in bridging intervals or investigate how variations in the timing of switching between anticoagulants might influence post-procedural outcomes.

In contrast to these prior studies, our retrospective observational analysis specifically evaluated the role of heparin-to-bivalirudin bridging time, an area that remains largely unexplored despite its clear mechanistic relevance. Building on the pathophysiological rationale that PCI disrupts atherosclerotic plaques, triggering platelet activation and thrombin generation (6, 23), and acknowledging the limitations of UFH—including unpredictable pharmacokinetics, need for frequent monitoring, and risks of heparin resistance or HIT (9, 10)—our results highlight that not only the choice of anticoagulant but also the timing of the transition may influence clinical outcomes.

Notably, we observed that patients with bridging intervals ≤30 min (Scenario A) were associated with a lower hospitalization rates compared with those with longer intervals, without an accompanying increase in bleeding or cardiovascular events. This finding suggests that maintaining a shorter transition window may help preserve more continuous and effective anticoagulation during the early periprocedural period—when PCI-related mechanical disruption of atherosclerotic plaque triggers a surge in platelet activation and thrombin generation (12). Ensuring adequate anticoagulation coverage during this critical phase may therefore contribute to improved early post-procedural clinical stability, consistent with prior evidence demonstrating that interruptions or insufficiency in antithrombotic therapy can increase peri-PCI ischemic risk (24). Cardiovascular events are clinical events that require a certain degree of severity. The advantage of short bridging time lies in preventing the development of critical states, which is manifested as a reduction in hospitalization rates. Hospitalization rate is a more sensitive indicator than cardiovascular events. In terms of mechanism, patients may experience discomfort, arrhythmia, or worsening heart failure due to a small thrombus load, microvascular dysfunction, or transient myocardial injury, and eventually be admitted to the hospital. In addition, although the platelet and APTT levels in the ≤30-min group were higher, the incidence of bleeding events did not increase. This indicates that within this time window, the anticoagulant overlap of heparin and bivalirudin is within a safe therapeutic window, achieving a balance between effective anticoagulation and bleeding risk. However, this association was not reproduced when the threshold was reduced to 20 min (Scenario B), and restricted cubic spline analysis revealed no dose-response relationship, indicating that the influence of bridging duration may be non-linear and that exceedingly short intervals may not provide incremental clinical benefit. The above results suggest that there is a critical time window for the anticoagulation status during the perioperative period, with the threshold possibly being around 30 min. This provides important guidance for clinical practice, indicating that operators do not need to immediately administer bivalirudin (such as within < 20 min) after stopping heparin, but can complete the transition within the 30-min safe time limit. However, due to the limited sample size of this study and the low event rate, the statistical power of the analysis of continuous variables may be insufficient, and thus it cannot be completely ruled out that there is a weak but real dose-response relationship. Future studies with a larger sample size are needed to confirm whether this relationship is threshold-based or reflects a more gradual continuous association.

Together, these observations expand upon previous evidence by demonstrating that while bivalirudin has a well-established bleeding advantage and predictable pharmacokinetic profile (20, 21, 25), the timing of transition from heparin to bivalirudin itself may represent an independent procedural parameter worth standardizing. Prior analyses of switching strategies during PCI, including the HORIZONS-SWITCH study and contemporary observational data (18, 19), have confirmed the feasibility and safety of transitioning from heparin to bivalirudin but did not examine the optimal interval between agents. Our results therefore complement existing trials by suggesting that an optimal bridging interval—potentially around 30 min—may enhance post-discharge outcomes, particularly hospitalization rates, without compromising safety. This interpretation is consistent with broader evidence emphasizing that periprocedural anticoagulation strategy, timing, and continuity constitute modifiable procedural variables that can meaningfully influence PCI outcomes (24). The findings of this study carry substantial economic implications. From a health economics perspective, the value of a treatment strategy must be assessed not only by direct drug acquisition costs but also by its broader impact on total healthcare expenditures, including downstream consequences such as re-hospitalization. Our results demonstrate that shortening the heparin-to-bivalirudin transition time to ≤30 min improves the cost-effectiveness of this sequential anticoagulation strategy, primarily by reducing costly re-hospitalizations, while preserving clinical safety. This advantage is especially salient in resource-constrained settings, where anticoagulation decisions are frequently dictated by budgetary constraints. Critically, the ≤30-min bridging protocol requires no additional pharmaceutical or procedural resources, making it both scalable and economically attractive. To robustly quantify its economic value across diverse healthcare systems, future prospective studies should incorporate formal cost-effectiveness analyses that comprehensively account for drug acquisition costs, procedure-related expenses, and hospitalization frequency and duration.

This study has several strengths, including the use of two complementary statistical approaches (logistic regression and GEE-based binomial regression), adjustment for multiple relevant confounders, exploration of two different bridging time thresholds to identify potential threshold effects, and comprehensive assessment of multiple clinical outcomes. The use of restricted cubic spline analysis to examine non-linear relationships provides additional insights beyond categorical threshold analyses.

However, several important limitations must be acknowledged. First, the relatively small sample size and event sparsity, limits statistical power and the precision of effect estimates, as reflected in the wide confidence intervals for some outcomes, which also limits the further expansion of subgroup analysis. Second, the retrospective, single-center design introduces potential for selection bias and limits generalizability to other healthcare settings with different patient populations or institutional protocols. In the future, prospective randomized trials are needed to further validate our conclusions. Third, the observed differences in baseline platelet counts and APTT among the various groups suggest the possibility of confounding factors caused by the severity of the disease or unmeasured patient characteristics. The operator might unconsciously tend to choose a shorter bridging time for patients who are considered to have a lower risk of thrombosis based on their preoperative baseline platelet count and APTT levels. To some extent, this could have led to the observed differences in hospitalization rates. Therefore, our results need to be interpreted with caution, and these baseline differences once again highlight the necessity of conducting randomized controlled trials to clarify the causal relationship and eliminate selection bias. Fourth, the decision regarding bridging time was made at operator discretion rather than through randomization, which may have introduced systematic bias. Fifth, our follow-up period was limited to six months to one year for hospitalization events, and longer-term outcomes remain unknown. Finally, we did not collect detailed information on the specific reasons for hospitalization, which limits our ability to determine whether the observed differences were driven by cardiovascular vs. non-cardiovascular causes or to elucidate the mechanisms underlying the association.

## Conclusions

In this study, shorter heparin-to-bivalirudin bridging times (≤30 min) were associated with significantly lower hospitalization rates compared to longer intervals, with no apparent increase in bleeding or other adverse events. However, this association was not observed with a 20-min threshold, and baseline differences between groups preclude definitive conclusions about causality. Large-scale prospective randomized trials are needed to validate these observations and establish optimal bridging protocols that balance clinical efficacy with cost-effectiveness in contemporary PCI practice.

## Data Availability

The raw data supporting the conclusions of this article will be made available by the authors, without undue reservation.
